# Effect of *Broussonetia papyrifera* silage on the serum indicators, hindgut parameters and fecal bacterial community of Holstein heifers

**DOI:** 10.1186/s13568-020-01135-y

**Published:** 2020-10-31

**Authors:** Hanchen Tian, Yiye Chen, Ni Zhu, Yongqing Guo, Ming Deng, Guangbin Liu, Yaokun Li, Dewu Liu, Baoli Sun

**Affiliations:** College of Animal Science, South China Agricultural University, 510642 Guangzhou, China

**Keywords:** *Broussonetia papyrifera* silage, Holstein heifers, Serum indicators, Hindgut fermentantion, Fecal bacterial community

## Abstract

This study investigated the effects of substitution of whole corn silage (WCS) with *Broussonetia papyrifera* silage (BPS) in different ratios on the serum indicators, hindgut fermentation parameters (pH, ammoniacal nitrogen, and volatile fatty acids), and fecal bacterial community of Holstein heifers. Sixteen heifers (8-month-old, 220 ± 30 kg) were randomly divided into four treatments according to different BPS substitution ratios of feed basis (0%, 25%, 50%, and 75%). The experiment consisted of a 7-day preliminary feeding period and a 30-day experimental period. On the last day of the trial, the blood samples were collected from caudal vein, and the feces samples were collected from rectum. With the increasing of BPS content, the concentration of malondialdehyde (MDA) and interleukin-1β (IL-1β) in serum decreased (*P* < 0.05), and the immunoglobulin A (IgA) and IL-4 content of serum increased (*P* < 0.05); and the hindgut pH value increased (*P* < 0.05). 16S rRNA sequencing found that the dominant phyla were *Firmicutes*, *Bacteroidetes*, and *Verrucomicrobia*; and the dominant genera were *Ruminococcaceae_UCG-005*, *Ruminococcaceae_UCG-010*, and *Rikenellaceae_RC9_gut_group*. Linear Discriminant Analysis Effect Size (LEfSe) analysis found 12 differential operational taxonomic units (OTUs) which have strong correlation with some serum and hindgut indicators, and have the potential to be used as biomarkers. Phylogenetic Investigation of Communities by Reconstruction of Unobserved States (PICRUSt) found that BPS have impacts on the pathways, such as carbohydrate transport and metabolism, and promotes amino acid transport and metabolism. To sum up, inclusion of BPS in heifer diets can affect serum anti-oxidant and immune indicators, fecal parameters, composition and function of fecal microorganisms in Holstein heifers.

## Introduction

With the increase of population and the decrease of arable land per capita, conventional feed such as forage and grain can no longer meet the need of animal husbandry (Dong et al. 2019; Zhai et al. 2020). In order to meet the demand of intensive animal production, it will be necessary to make novel and unconventional feed resources available (Araújo et al. 2019). Unconventional feed materials are those that are not commonly used in formulations or have little research on their nutritional properties and feeding value, but have high yield and wide distribution and varieties. Currently, unconventional feed like Chinese jujube meal, pomegranate residue, orange leaves, olive leaves have been reported (Xie et al. 2018; Fernández et al. 2019; Hukerdi et al. 2019; Khorsandi et al. 2019). The above studies showed that those unconventional feed can promote the production performance of livestocks to some extent with no adverse effect.

*Broussonetia papyrifera* (Paper mulberry), a deciduous tree of *Moraceae* family, is widely distributed in China, Japan and other Asian countries (Yao et al. 2017). Many biologically active compounds contained in *B. papyrifera*, such as flavonoids, lignans, polysaccharides, and terpenoids might have antimicrobial, anti-inflammatory, and antioxidant properties and can reduce the growth of tumors (Mei et al. 2009; Sohn et al. 2010; Wang et al. 2010; Xu et al. 2010; Sun et al. 2012; Guo et al. 2013; Han et al. 2016). For this reason, *B. papyrifera* is already widely used in the pharmaceutical industry. Due to its high-quality fiber, *B. papyrifera* is also used as important raw material for the production of paper (Peng et al. 2019). *B. papyrifera* has advantages of rapid growth, strong adaptability and disease resistance, and high protein content (approximate 18–22%, including both leaves and stems) (Peng et al. 2019), which makes it a potential candidate for new feed resourse.

Intestinal bacteria, which are diverse and abundant, play vital parts in maintaining the homeostasis of the digestive system and the function of the immune system (Liu et al. 2019). Intestinal bacteria have two main functions: converting nutrients into energy and resisting the invasion of pathogenic microorganisms (Guarner 2007). The stability of intestinal flora is directly related to animal physical condition, thus affecting production performance. In ruminant, intestinal flora is influenced by feed ingredients. Xie et al. (2020) fed Holstein heifers by substituting different proportions (0%, 5%, and 10%) of whole corn silage (WCS) with herbal tea residue, finding that the microbial composition of the three treatments was significantly different. Moreover, Sun et al. (2017) fed Holstein cows different percentages of ensiled *Moringa oleifera* and found a strong correlation between the presence of *Akkermansia* and *Prevotella* in total milk yield and milk protein, which indicates that some bacterial groups could be associated with enhanced milk production performance.

WCS is the most common forage used in ruminant production in China. However, due to some constraints (climate, soil type, etc.), the yield of WSC and other common forages in some regions in China could meet the requirement of local ruminant production. As a new feed material, *B. papyrifera* has been applied in aquaculture, pig, poultry, and ruminant production. Nevertheless, studies on the effects of feeding *B. papyrifera* silage to Holstein cows are still limited. In the present study, effects of substitution of WCS with different proportions of *B. papyrifera* silage (BPS) on the serum anti-oxidant and immune indicators; hindgut fermentation parameters including pH value, ammoniacal nitrogen (NH_3_-N), and volatile fatty acid (VFA); and fecal bacterial community of Holstein heifers were studied, providing a reference for the application of *B. papyrifera* in production. In depth, we analyzed the correlation between bacterial community and various serum and hindgut indicators, and predicted the function of bacterial community, in order to reveal the cause of differences caused by feed on the microbial level.

## Materials and methods

All experimental procedures used in this study were approved by the Committee of Animal Experiments of South China Agricultural University (No. 201004152).

### Experimental materials

*B. papyrifera* silage was purchased from a feed company (Heyuan, GD, China). Hybrid *B. papyrifera* was cut off when it reached a height of 1–1.5 m, and the trunk and branches were removed by a straw chopper, leaving the front 20–30 cm thin branches and leaves, and cutting them into 1 cm per segment. After air drying moderately, the mince, including leaves and twigs, was made into silage via stretch-film-wrapped silage technology (30.00 ± 5.00 kg/bale). Then the packaged silages were preserved in a dry and room-temperature indoor environment for 60 days.

### Experimental animals

This experiment was carried out in a commercial dairy farm (Yangjiang, GD, China) and adopted a completely randomized block, including a 7-day preliminary feeding period and a 30-day experimental period. Sixteen healthy 8-month-old Holstein heifers (220 ± 30 kg) were randomly assigned to four treatments. In the four groups, the substitution ratios (feed basis) of BPS for WCS in diets was 0% (T0), 25% (T25), 50% (T50), and 75% (T75), respectively. The total mixed ration (TMR) were formulated based on Chinese feeding standards (China Standard NY/T34, 2004). The nutrient composition of WCS, BPS, and TMR were analyzed. Thereinto, dry matter (DM), crude protein (CP), ether extract (EE) were measured according to Association of Official Analytical Chemists (AOAC 2002); neutral detergent fiber (NDF), acid detergent fiber (ADF), and hemicellulose (HC) were tested based on Van Soest et al. (1991); calcium (Ca) was measured via EDTA complexometric titration method; phosphorus (P) was determined via vanadium molybdate yellow colorimetric method. The nutrient content of WCS and BPS were shown in Table 1. The ingredient and nutrient content of TMRs were shown in Table 2. Heifers were fed twice per day (10:00 and 16:00), and had *ad libitum* access to feed and water throughout the experimental period. The heifers were kept in a wide-open free-stall barn with natural ventilation and the excrement was cleaned artificially every day. The cowshed took natural light during the day and artificial light at night, in order to ensure all-day illumination. The blood and feces samples were collected at 4 h after morning feeding on the last day of experimental period. The blood (approximately 20 mL) was collected from caudal vein; after centrifugation (4000 r/min, 15 min), the upper serum was separated and stored at − 20 ℃. The feces samples were collected from rectum, approximately 2 g of each samples were stored at -80℃ for the determination of microbial flora, another 10 g was taken and added into 20 mL of double steamed water for the determination of fecal parameters, including pH, NH_3_-N, and VFAs.

**Table 1:** Chemical composition of WCS and BPS

Item	WCS	BPS
DM	30.50	30.86
CP (%/DM)	10.70	14.34
EE (%/DM)	3.30	2.33
NDF (%/DM)	60.10	49.23
ADF (%/DM)	38.90	31.21
Ash (%/DM)	11.37	11.52

WCS, whole corn silage; BPS, *Broussonetia papyrifera* silage; DM, dry matter; CP, crude protein; EE, ether extract; NDF, neutral detergent fiber; ADF, acid detergent fiber

**Table 2:** Ingredients and chemical composition of the diets

Item^a^	Diets treatment^b^			
	T0	T25	T50	T75
Ingredient (as fed-basis %)				
Hay	13.07	13.14	13.22	13.29
Soybean meal	3.40	3.07	2.91	2.66
Salt	0.17	0.18	0.18	0.18
Mineral premix^c^	0.09	0.09	0.09	0.09
Indian meal	2.61	2.41	1.98	1.77
DDGS	2.26	2.28	2.29	2.30
Brewer's grains	8.71	8.76	8.81	8.86
BPS	0.00	17.52	35.26	53.14
WCS	69.69	52.55	35.26	17.71
Nutrient content				
CP (%DM)	13.44	14.92	13.30	15.44
EE (%DM)	2.10	1.89	1.91	1.94
NDF (%DM)	51.48	50.46	50.14	50.34
ADF (%DM)	30.02	31.32	30.20	30.78
HC (%DM)	21.46	19.14	19.94	19.56
Ca (%DM)	0.78	0.79	0.80	0.78
P (%DM)	0.45	0.43	0.43	0.44
RUP (%CP)	42.27	42.65	43.22	43.59
ME (mcal/kg DM)	1.86	1.87	1.88	1.89

^a^DDGS, distillers dried grains with solubles; BPS, *Broussonetia papyrifera* silage; WCS, whole corn silage; DM, dry matter; CP, crude protein; EE, ether extract; NDF, neutral detergent fiber; ADF, acid detergent fiber; HC, hemicellulose; RUP, rumen undegradable protein; ME, metabolizable energy

^b^T0, BPS replaces 0% of WCS; T25, BPS replaces 25% of WCS; T50, BPS replaces 50% of WCS; T75, BPS replaces 75% of WCS. BPS, *Broussonetia papyrifera* silage; WCS, whole corn silage

^c^Mineral premix provided the following per kg of concentrate, vitamin A, 120–180 KIU; vitamin D3, 40–60 KIU; vitamin E, ≥ 1,102 mg; Cu, 459–613 mg; Mn, 918-1,225 mg; Zn, 1,840-2,455 mg; Se, 12–18 mg; I, 22.5–30 mg; Co, 5.4–7.34 mg

### Serum indicators determination

Serum antioxidant indicators, including malondialdehyde (MDA), superoxide dismutase (SOD), glutathione peroxidase (GSH-Px), catalase (CAT), and total antioxidant capacity (T-AOC), were tested via a commercial kit (Nanjing Jiancheng Bio-Engineering Co. Ltd., Nanjing, China) (Peng et al. 2020). Serum immune indicators, including immunoglobulin A (IgA), interleukin 1β (IL-1β), IL-2, IL-4, IL-6, IL-10, and IL-17, were tested via enzyme-linked immunosorbent assay (ELISA) kit (Jiangsu Jingmei Biotechnology Co. Ltd., Yancheng, China) (Dong et al. 2019). The detailed operating steps of kits were showed in manufacture’s protocols in detail.

### Hindgut fermentation parameters determination

Approximately 10 g of each fresh feces sample was mixed with 20 mL distilled water, and then shaked up and centrifuged (5400 rpm × 10 min). The supernatant was collected to test pH-value and the content of NH_3_-N and VFAs, including acetic acid (AA), propionic acid (PA), isobutyric acid (IBA), butyric acid (BA), isovaleric acid (IVA), and valeric acid (VA).

The determination of NH_3_-N content were according to the method of Broderick and Kang (1980). Briefly, a spectrophotometer (UV-2600, Unico, Shanghai) was used for colorimetry, and the standard curve was obtained according to OD value of standard ammonia solution. The prepared phenol reagent and sodium hypochlorite reagent were successively added to the supernatant mentioned above, after water bath, OD value of the solution was calculated at the wavelength of 630 nm.

The determination of volatile fatty acids (VFA) was based on the method described by Erwin et al. (1961), and the column selection was adjusted and some chromatographic operating conditions were optimized. The chromatographic column was HP-INNOWax capillary column and set to constant flow mode, flow: 2.0 mL/min, mean linear velocity: 38 cm/s. The parameters of gas chromatograph were set as follows: carrier gas, N_2_; injection volume, 0.6 µL; injection temperature, 220℃; split ratio, 40:1. In this research, 2-ethyl BA (2-EB) was selected as the internal standard. Based on the established integral parameter and correction curve, the content of each component of the unknown sample was obtained by internal standard calculation method.

### Bacterial community analysis

The total DNA of bacterial community was extracted via E.Z.N.A.® DNA Kit (Omega Biotek, Norcross, GA, USA) according to the specification. The ABI GeneAmp®9700 PCR Amplifier (ABI, Carlsbad, CA, USA) was adopted to amplify the 16S rRNA V3-V4 regions. The primers were designed as follows: forward primer, 338F (ACTCCTACGGGAGGCAGCAG); and reverse primer, 806R (GGACTACHVGGGTWTCTAAT) (He et al. 2019). PCR system was as follows: 5 × FastPfu buffer, 4 µL; 2.5 mM dNTPs, 2 µL; forward primer (5 µM), 0.8 µL; reverse primer (5 µM), 0.8 µL; FastPfu polymerase, 0.4 µL; bovine serum albumin (BSA), 0.2 µL; template DNA, 10 ng; ddH_2_O, fill to 20 µL. The reaction was performed under the following condition: 95 ℃ for 3 min; 95 ℃ for 30 s, 55 ℃ for 30 s, 72 ℃ for 45 s for 27 cycles; 72 ℃ for 10 min. After purification, detection, and quantification, the PCR products were sequenced via Illumina Miseq platform (Majorbio Bio-Pharm Technology Co. Ltd., Shanghai, China). After sequencing, the raw data was analyzed according to Sun et al. (2017).

### Statistical analysis

Serum and fecal parameters were analyzed using SAS 9.4 software (SAS Institute Inc., Cary, NC, USA). Briefly, INFLUENCE statement was used for outlier elimination; GLM procedure was invoked for data processing; LSMEANS statement was adopted to calculate least squares mean; Tukey method was used for multi-comparison. The model used for data processing is: Y_ij_ = µ + T_i_ + ε_ij_, thereinto, Y_ij_ is the dependent variable value of the test heifers in different treatments; µ is the overall mean; T_i_ is the diets treatment effects; ε_ij_ is the random error. Orthogonal polynomial contrasts (linear, quadratic, and cubic) were used to analyze the effects of the different BPS inclusion levels on the serum and fecal parameters. The experimental data were presented in tables with mean value and standard error of mean (SEM), *P* < 0.05 indicated significant differences.

Linear Discriminant Analysis (LDA) Effect Size (LEfSe) analysis was performed via an online tool (http://huttenhower.sph.harvard.edu/galaxy/) in order to obtain differential operational taxonomic units (OTU). Principal component analysis (PCA) and Adonis analysis were performed via vegan package in R 4.0.3 software. The correlation analyses between differential OTU and various indicators were carried out via WGCNA package in R 4.0.3 software (Sun et al. 2017); and the results showed P-Value and correlation coefficient (C_XY_). Clusters of Orthologous Groups (COG) functional annotation and Kyoto Encyclopedia of Genes and Genomes (KEGG) sample abundance statistics were computed via Phylogenetic Investigation of Communities by Reconstruction of Unobserved States (PICRUSt) on Majorbio (Shanghai, China) online platform (http://www.i-sanger.com).

## Results

### Serum indicators and hindgut fermentation parameters

The MDA content of T50 declined linearly (*P* < 0.05) compared to T0 (Table 3), no significant differences were found between T25 and T75. Other antioxidant indicators had no significant differences in the four treatments. For serum immune indicators (Table 2), compared with T0, the IgA content of T25, T50, and T75, the IL-4 content of T75 increased (linear, *P* < 0.05); the IL-1β content of T50 and T75 decreased (linear, *P* < 0.05). The IL-10 content of T75 was significantly lower than that in T25 (*P* < 0.05). For hindgut fermentation parameters (Table 4), the pH value of T25 was higher than that in T0 (*P* < 0.05); the NH_3_-N content of T50 and T75 increased compared with T25 (*P* < 0.05), there was no difference between T0 and the other three group treated with BPS. Other indicators did not differ significantly between the four treatments.

**Table 3:** Effects of BPS on the serum indicators of Holstein heifers (n = 4)

Item^1^	Diets treatment^2^				SEM^3^	P-value		
	T0	T25	T50	T75		Linear	Quadratic	Cubic
Serum anti-oxidant indicators								
MDA (mmol/L)	2.61^a^	2.24^ab^	1.79^b^	2.34^ab^	0.20	0.018	0.675	0.242
SOD (U/mL)	88.90	88.93	87.48	85.46	2.68	0.332	0.708	0.942
GSH-Px (U/mL)	58.45	50.31	44.50	43.63	7.40	0.134	0.631	0.941
CAT (U/mL)	0.47	0.50	0.37	0.42	0.05	0.286	0.827	0.161
T-AOC (mmol/L)	0.46	0.41	0.43	0.41	0.02	0.128	0.513	0.221
Serum immune indicators								
IgA (g/L)	0.11^b^	0.13^a^	0.14^a^	0.14^a^	0.01	0.019	0.224	0.774
IL-1β (ng/L)	46.86^a^	45.61^a^	38.77^b^	40.08^b^	2.20	0.011	0.572	0.210
IL-2 (ng/L)	292.84	290.91	281.16	252.76	15.47	0.054	0.974	0.718
IL-4 (ng/L)	77.99^b^	83.62^b^	93.19^ab^	106.34^a^	5.21	0.001	0.482	0.988
IL-6 (ng/L)	11.39	10.36	10.46	9.88	0.87	0.263	0.796	0.657
IL-10 (ng/L)	43.93^ab^	48.03^a^	43.90^ab^	41.44^b^	1.90	0.122	0.483	0.558
IL-17 (ng/L)	56.05	51.56	45.33	52.55	3.29	0.254	0.097	0.341

^1^MDA, malondialdehyde; SOD, superoxide dismutase; GSH-Px, glutathione peroxidase; CAT, catalase; T-AOC, total antioxidant capacity; Ig, immunoglobulin A; IL, interleukin

^2^T0, BPS replaces 0% of WCS; T25, BPS replaces 25% of WCS; T50, BPS replaces 50% of WCS; T75, BPS replaces 75% of WCS. BPS, *Broussonetia papyrifera* silage; WCS, whole corn silage. Different letters mean significant difference

^3^SEM, standard error of mean

**Table 4:** Effects of BPS on the hindgut fermentation parameters of Holstein heifers (n = 4)

Item^1^	Diets treatment^2^				SEM^3^	P-value		
	T0	T25	T50	T75		Linear	Quadratic	Cubic
pH	6.79^a^	7.18^b^	7.02^ab^	7.05^bc^	0.10	0.174	0.091	0.146
AA (mmol/L)	12.26	11.48	11.34	11.00	1.24	0.470	0.858	0.887
PA (mmol/L)	2.51	2.17	2.28	2.17	0.26	0.432	0.655	0.584
IVA (mmol/L)	0.34	0.35	0.39	0.37	0.03	0.304	0.566	0.481
BA (mmol/L)	1.14	0.87	0.79	0.98	0.16	0.428	0.173	0.906
IVA (mmol/L)	0.28	0.29	0.32	0.31	0.02	0.212	0.711	0.731
VA (mmol/L)	0.20	0.18	0.18	0.21	0.03	0.830	0.286	0.948
NH_3_-N (mg/dL)	5.52^abc^	4.47^a^	5.40^b^	5.98^bc^	0.45	0.250	0.093	0.291

^1^AA, acetic acid; PA, propionic acid; IBA, isobutyric acid; BA, butyric acid; IVA, isovaleric acid; VA, valeric acid; NH_3_-N, ammoniacal nitrogen

^2^T0, BPS replaces 0% of WCS; T25, BPS replaces 25% of WCS; T50, BPS replaces 50% of WCS; T75, BPS replaces 75% of WCS. BPS, *Broussonetia papyrifera* silage; WCS, whole corn silage. Different letters mean significant difference

^3^SEM, standard error of mean

### 16S rRNA gene sequencing and analysis

**Fig. 1:**
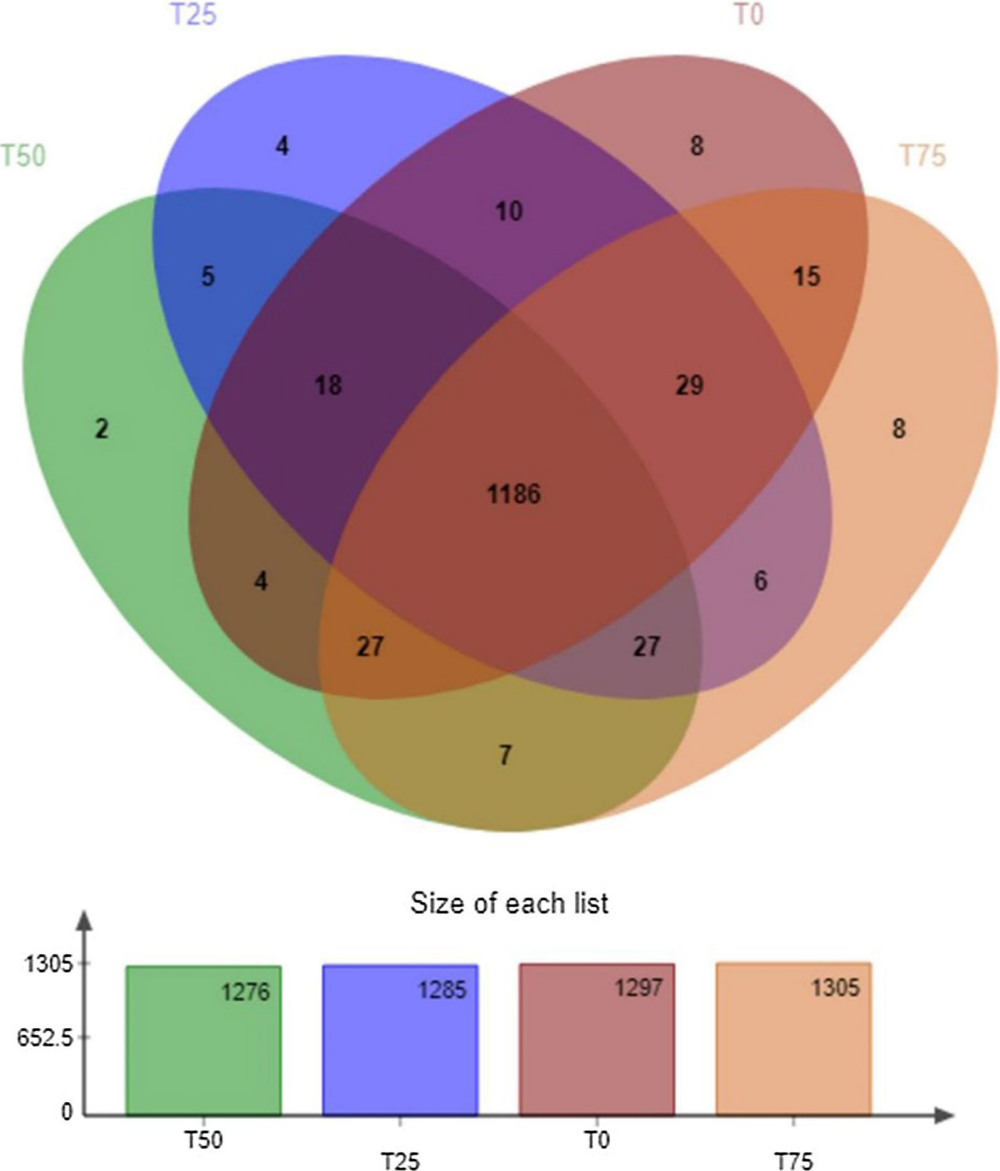
Venn map of operational taxonomic units (OTU) in the four treatment. T0: BPS replaces 0% of WCS; T25: BPS replaces 25% of WCS; T50: BPS replaces 50% of WCS; T75: BPS replaces 75% of WCS. BPS, *Broussonetia papyrifera* silage; WCS, whole corn silage

Miseq high-throughput sequencing was performed on the V3–V4 region of 16S rRNA, and a total of 424,032 effective sequences were obtained, 26,502 for each sample. According to high-throughput sequencing results, cluster analysis of OTUs was conducted, and a total of 15 phyla, 26 classes, 39 orders, 67 families, 185 genera, 341 species, and 1356 OTUs were obtained. The Venn diagram of this study was shown in Fig. 1. As shown in the figure, the number of OTU that all the four treatments possess was 1186, and it accounted for 87.46% of the total amount.

**Fig. 2:**
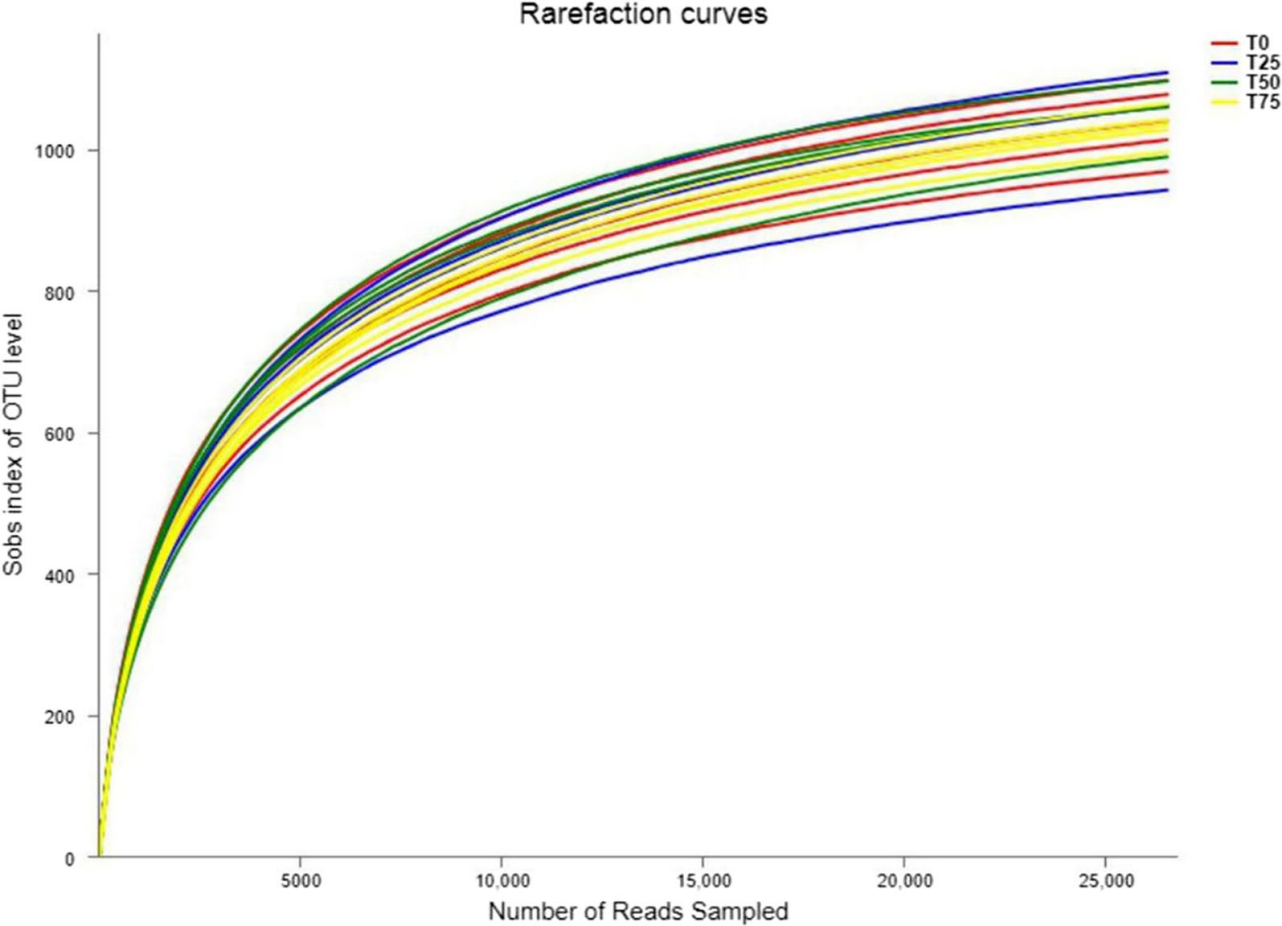
Rarefaction curves of feces samples

**Fig. 3:**
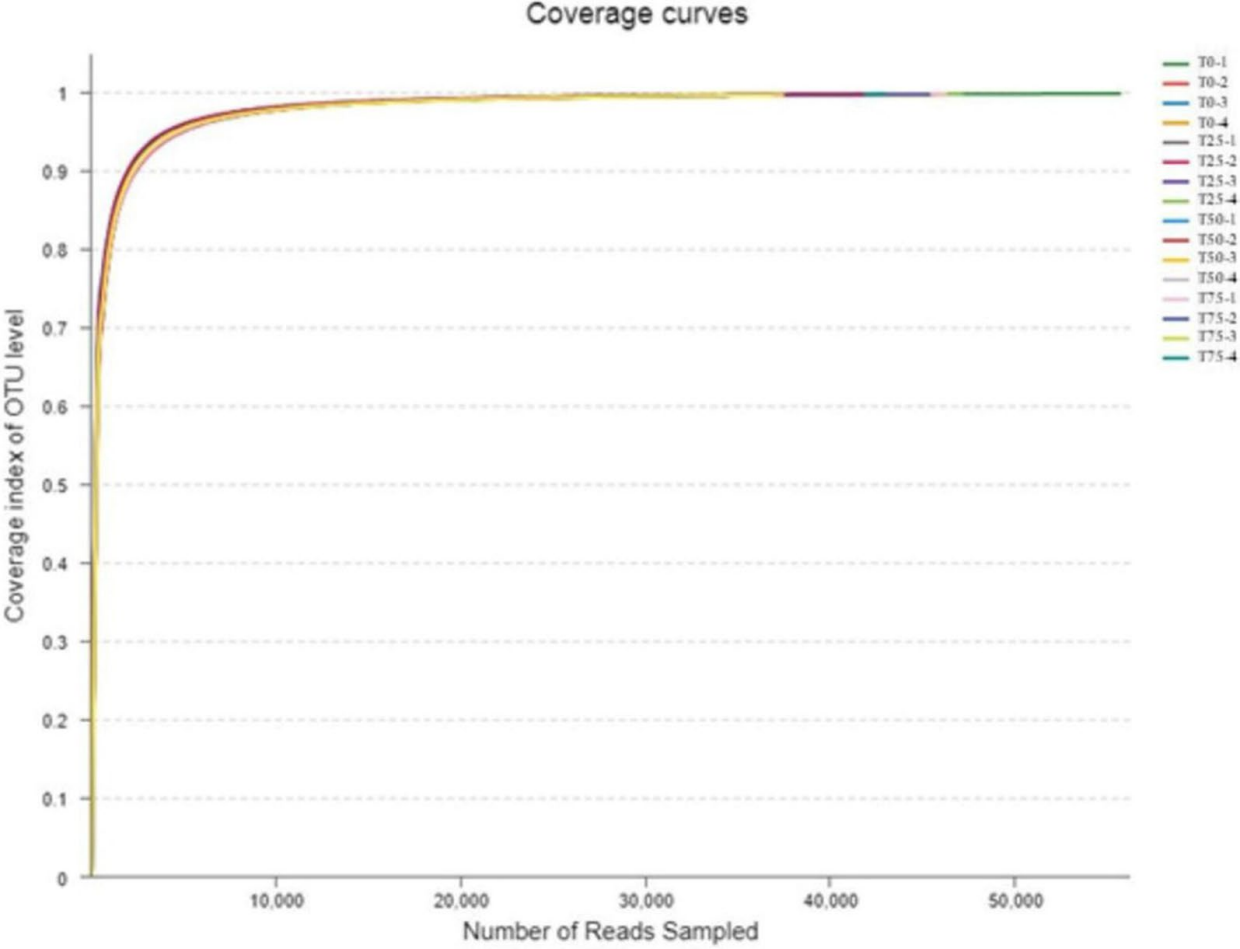
Coverage curves of feces samples

**Fig. 4:**
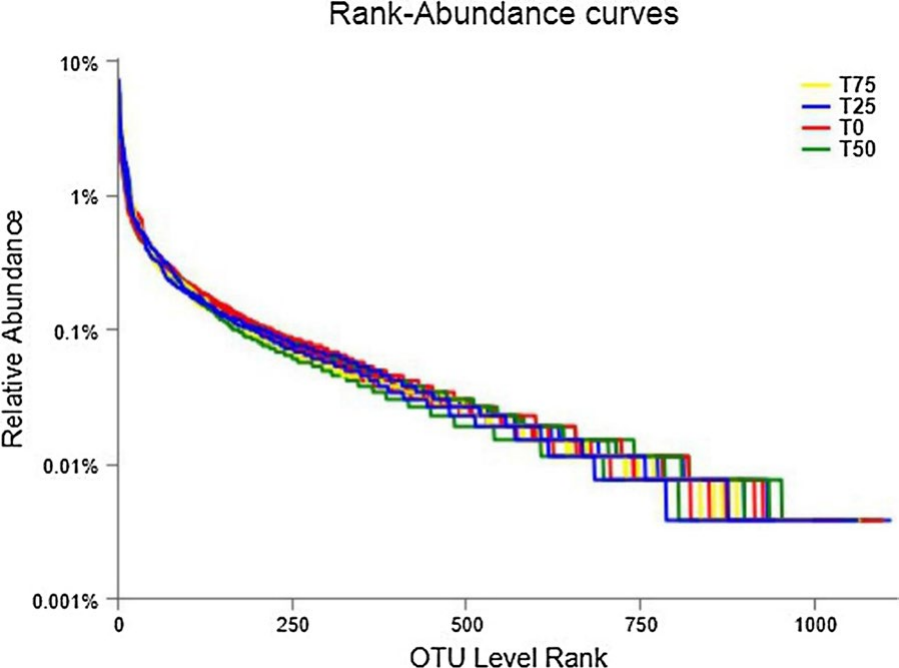
Rank-abundance curves of feces samples

The rarefaction curve is commonly used to reflect the sequencing depth and coverage of test samples. Figure 2 shows that the rarefaction curve of 16 samples sequenced in this test did not enter the plateau phase when the number of sequencing reads reached 25,000, indicating that the sequencing data volume cannot absolutely represent all OTUs in the bacterial community of feces, and it is still possible to find new OTUs by increasing the sequencing data volume. However, the growth rate of rarefaction curve has slowed, suggesting a sufficient level of species richness. According to the coverage curve in Fig. 3, when the number of sequencing reads reached 10,000, the coverage reached 97%, and when the number of sequencing reads continued to grow to 25,000, the coverage was close to 100%.It shows that the depth and coverage of sequencing data are reasonable and the measured data can be used for subsequent analysis. The rank-abundance curve of fecal samples was shown in Fig. 4. It can be seen from the figure that the microbial diversity of the 4 treatments is similar.

**Fig. 5:**
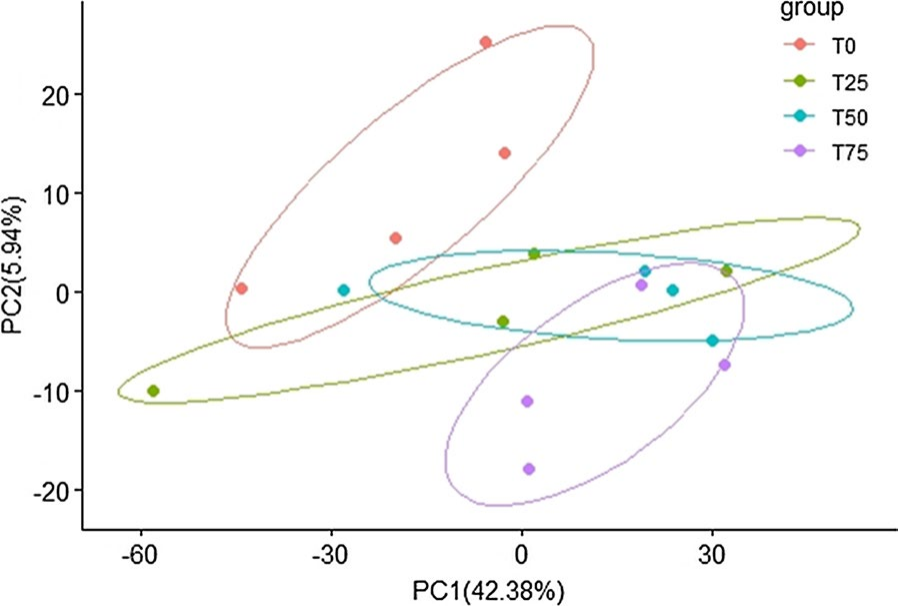
Principal component analysis of the four treatment. T0: BPS replaces 0% of WCS; T25: BPS replaces 25% of WCS; T50: BPS replaces 50% of WCS; T75: BPS replaces 75% of WCS. BPS, *Broussonetia papyrifera* silage; WCS, whole corn silage

For α-diversity, Shannon index, Ace index, and Chao index were calculated and the results were shown in Table 5. As we can see from the table, there were no significant differences in the above three indexes, indicating BPS had no remarkably effect on the diversity and abundance of fecal bacteria community. For β-diversity, the results of PCA and Adonis were shown as Fig. 5; Table 6. As we can see in Fig. 5, the separation between Treatment T0 and other treatments is obvious. Table 6 showed that there are significant changes between Treatments T0 and T75 (*P* < 0.05).

**Table 5:** Effects of BPS on the α-diversity of hindgut bacterial community of Holstein heifers (n = 4)

Item	Diets treatment^a^				SEM^b^	P-value		
	T0	T25	T50	T75		Linear	Quadratic	Cubic
Shannon	5.64	5.58	5.48	5.56	0.06	0.603	0.227	0.155
Ace	1144.55	1145.67	1143.72	1153.51	24.18	0.794	0.860	0.978
Chao	1150.60	1154.06	1147.21	1165.92	25.20	0.647	0.767	0.964

^a^T0, BPS replaces 0% of WCS; T25, BPS replaces 25% of WCS; T50, BPS replaces 50% of WCS; T75, BPS replaces 75% of WCS. BPS, *Broussonetia papyrifera* silage; WCS, whole corn silage

^b^SEM, standard error of mean

**Table 6:** Adonis analysis of the four treatments

Items^a^	R^2^	P value
T0/T25	0.1256	0.581
T0/T50	0.2196	0.085
T0/T75	0.2553	0.027
T25/T50	0.1381	0.422
T25/T75	0.1384	0.475
T50/T75	0.1440	0.360

^a^T0, BPS replaces 0% of WCS; T25, BPS replaces 25% of WCS; T50, BPS replaces 50% of WCS; T75, BPS replaces 75% of WCS. BPS, *Broussonetia papyrifera* silage; WCS, whole corn silage

**Fig. 6:**
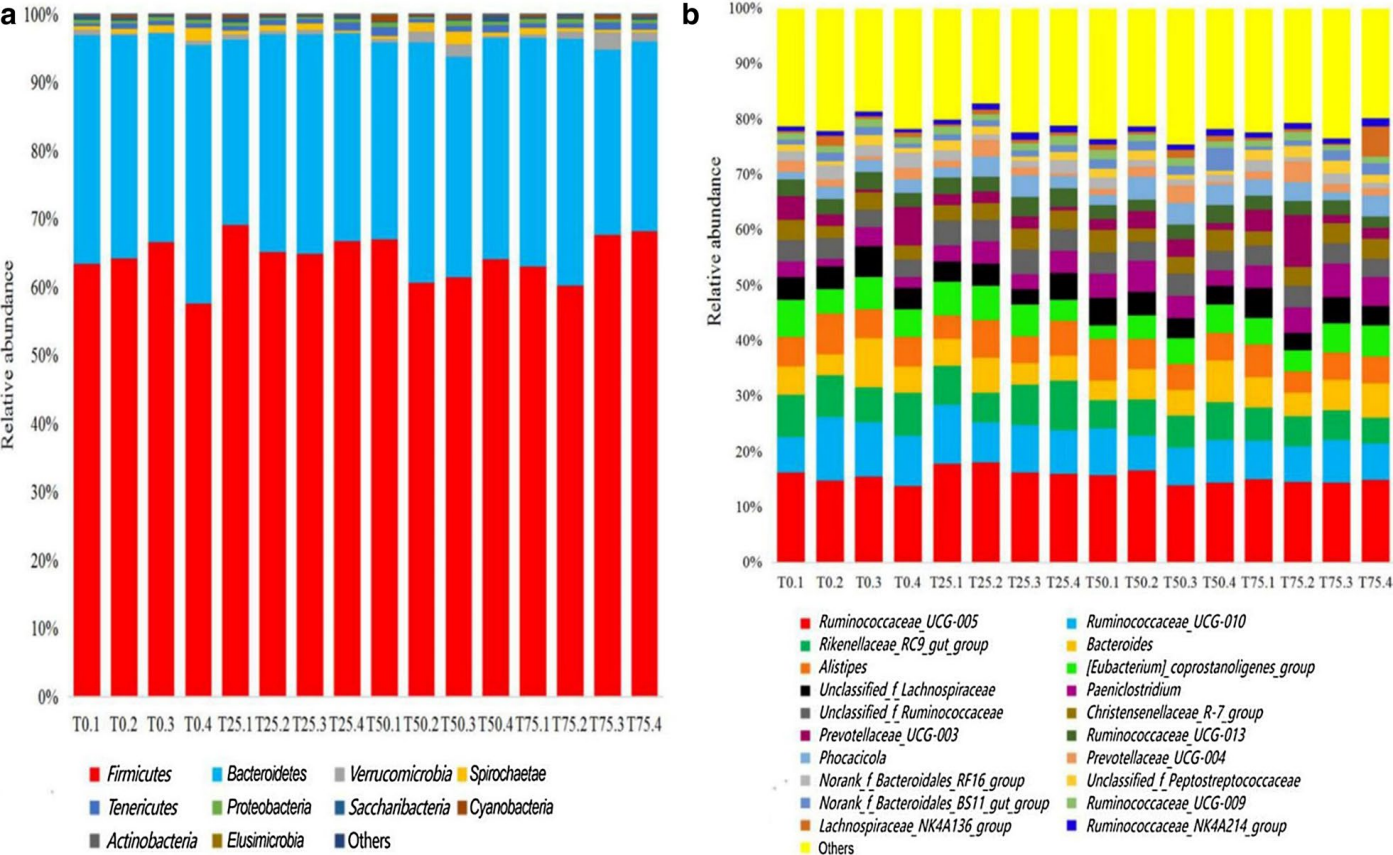
Relative abundance of fecal bacterial community on the phylum and genus level. Figure 6a is the relative abundance on the phylum level; Fig. 6b is the relative abundance on the genus level. T0: BPS replaces 0% of WCS; T25: BPS replaces 25% of WCS; T50: BPS replaces 50% of WCS; T75: BPS replaces 75% of WCS. Numbers 1, 2, 3 and 4 in names of samples refer to individual heifers per set of treatment. BPS, *Broussonetia papyrifera* silage; WCS, whole corn silage

The composition of bacterial community on the phylum and genus levels were shown in Fig. 6. *Firmicutes* and *Bacteroidetes* were the two most dominant phyla, accounting for more than 95% of the bacterial community. On the genus level, in different treatments, *Ruminococcaceae_UCG-005*, *Ruminococcaceae_UCG-010*, and *Rikenellaceae_RC9_gut_group* were always the first three dominant genera. With the increasing of BPS content, some phyla, including *Firmicutes*, *Bacteroidetes*, *Tenericutes*, *Proteobacteria*, and *Verrucomicrobia*, and some genera, including *Paeniclostridium*, *Phocaeicola*, and *Norank_f_bacteroidales_BS11_gut_group*, became more abundant; while other genera, such as *Ruminococcaceae_UCG-013*, *Ruminococcaceae_UCG-010*, and *Ruminococcaceae_UCG-009* decrease.

### Correlation between differential OTUs and various indicators

**Fig. 7:**
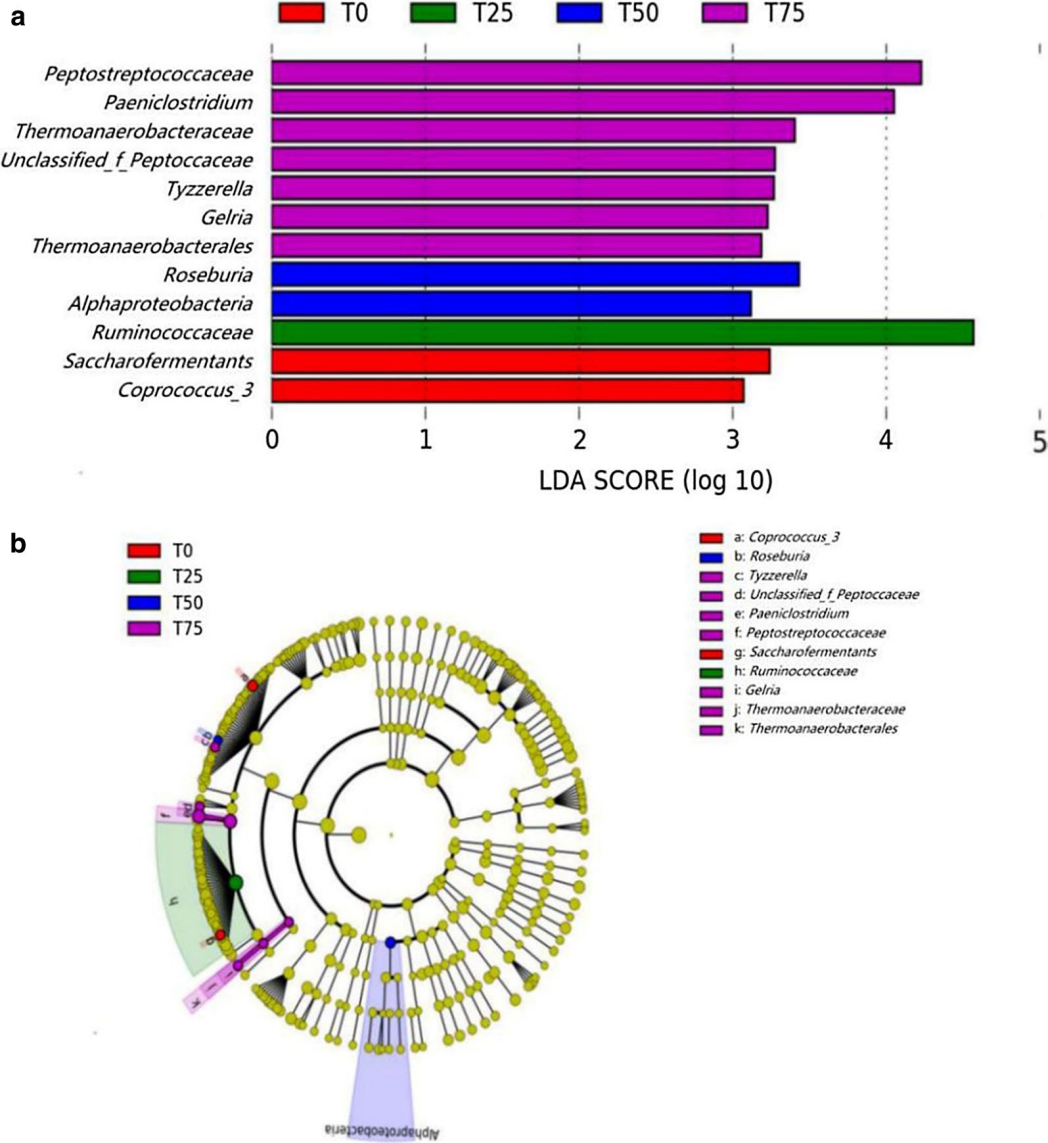
Comparison of microbial variations using the LEfSe online tool. T0: BPS replaces 0% of WCS; T25: BPS replaces 25% of WCS; T50: BPS replaces 50% of WCS; T75: BPS replaces 75% of WCS. Numbers 1, 2, 3 and 4 in names of samples refer to individual heifers per set of treatment. BPS, *Broussonetia papyrifera* silage; WCS, whole corn silage

LDA score represented the influence of significantly different OTUs. The OTU, which its LDA score was greater than the set point, can be regarded as statistically significant biomarker. In the present study, the set point was 2, and 12 differential OTUs (Fig. 7), including *Peptostreptococcaceae* (family), *Paeniclostridium* (genus), *Thermoanaerobacteraceae* (family), *Unclassified_f_peptoccaceae* (genus), *Tyzzerella* (genus), *Gelria* (genus), *Thermoanaerobacterales* (order), *Roseburia* (genus), *Alphaproteobacteria* (class), *Ruminococcaceeae* (family), *Saccharofermentants* (genus), and *Coprococcus_3* (genus) were obtained. Figure 7a was the LDA score distribution histogram, the different colors represented their respective groups; and the length represented the LDA score, which is the degree of influence of the differential OTUs between the four treatments. Figure 7b was the cladogram, the circle radiating from the inside to the outside represented the classification level from phylum to genus, and the diameter of the circle represented the relative abundance. The OTUs with no significant difference were uniformly colored yellow, and the differential OTUs followed the treatment.

**Fig. 8:**
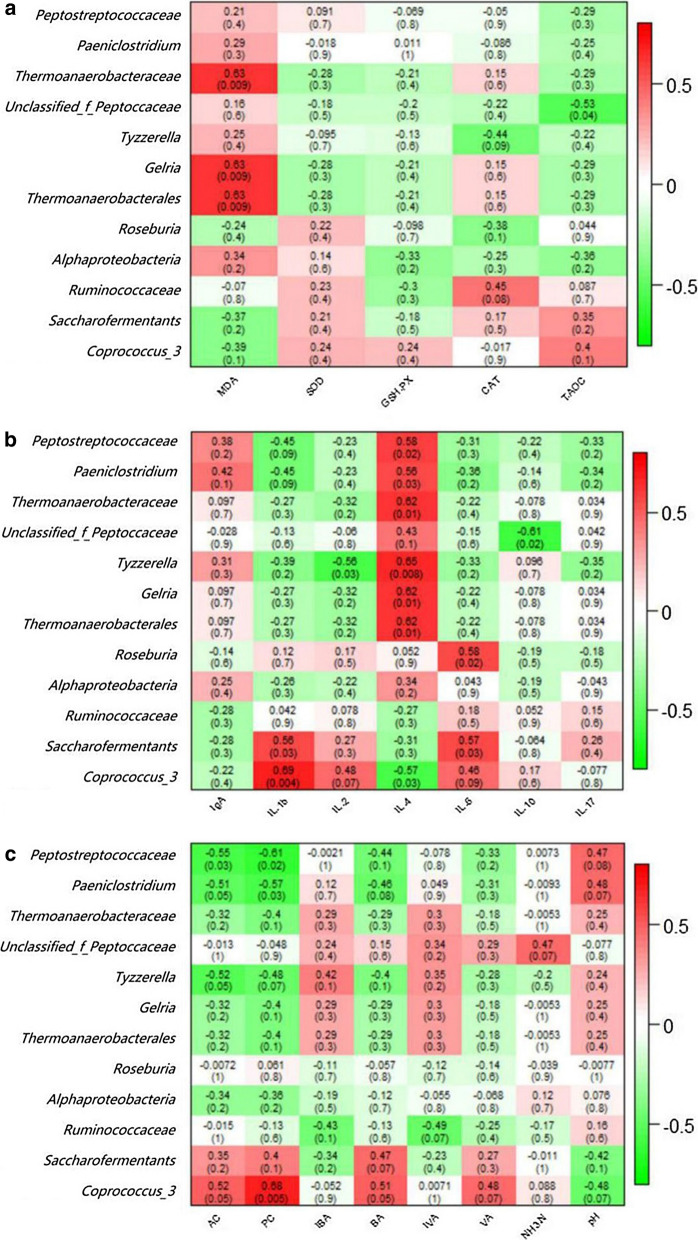
Correlation analyses of differential OTUs with serum anti-oxidant indicators (**a**), serum immune indicators (**b**), and hindgut fermentation parameters (**c**). Each cell contains Pearson correlation coefficient and P-value (within brackets). **a** MDA, malondialdehyde; SOD, superoxide dismutase; GSH-Px, glutathione peroxidase; CAT, catalase; T-AOC, total antioxidant capacity. **b** Ig, immunoglobulin; IL, interleukin. **c** AA, acetic acid; PA, propionic acid; IBA, isobutyric acid; BA, butyric acid; IVA, isovaleric acid; VA, valeric acid; NH_3_-N, ammoniacal nitrogen

In this study, differential OTUs were performed correlation analysis with serum antioxidant and immune indicators, and hindgut fermentation parameters (Fig. 8). For serum antioxidant indicators, *Thermoanaerobacteraceae*, *Thermoanaerobacterales*, and *Gelria* were positively correlated with MDA (C_XY _= 0.63, P = 0.009; C_XY _= 0.63, P = 0.009; C_XY _= 0.63, P = 0.009, respectively); *Unclassified_f_peptoccaceae* was negatively correlated with CAT (C_XY _= − 0.53, P = 0.04). For serum immune indicators, *Peptostreptococcaceae*, *Paeniclostridium*, *Thermoanaerobacteraceae*, *Gelria*, and *Thermoanaerobacterales* had positive correlation with IL-4 (C_XY _= 0.58, P = 0.02; C_XY _= 0.56, P = 0.03; C_XY _= 0.62, P = 0.01; C_XY _= 0.62, P = 0.01; C_XY _= 0.62, P = 0.01, respectively); *Roseburia* had positive correlation with IL-6 (C_XY _= 0.58, P = 0.02); *Saccharofermentants* had positive correlation with IL-1β and IL-6 (C_XY _= 0.56, P = 0.03; C_XY _= 0.57, P = 0.03, respectively); *Coprococcus_3* had positive correlation with IL-1β (C_XY _= 0.69, P = 0.004); *Unclassified_f_peptoccaceae* had negative correlation with IL-10 (C_XY _= − 0.61, P = 0.02); *Tyzzerella* had negative correlation with IL-2 (C_XY _= − 0.56, P = 0.03); *Coprococcus_3* had negative correlation with IL-4 (C_XY _= − 0.57, P = 0.03). For hindgut fermentation parameters, *Coprococcus_3* was positively correlated with PA (C_XY _= 0.68, P = 0.005); *Peptostreptococcaceae* was negatively correlated with AA and PA (C_XY _= − 0.55, P = 0.03; C_XY _= − 0.61, P = 0.02); *Paeniclostridium* was negatively correlated with PA (C_XY _= − 0.57, P = 0.03).

### 16S function prediction

**Fig. 9:**
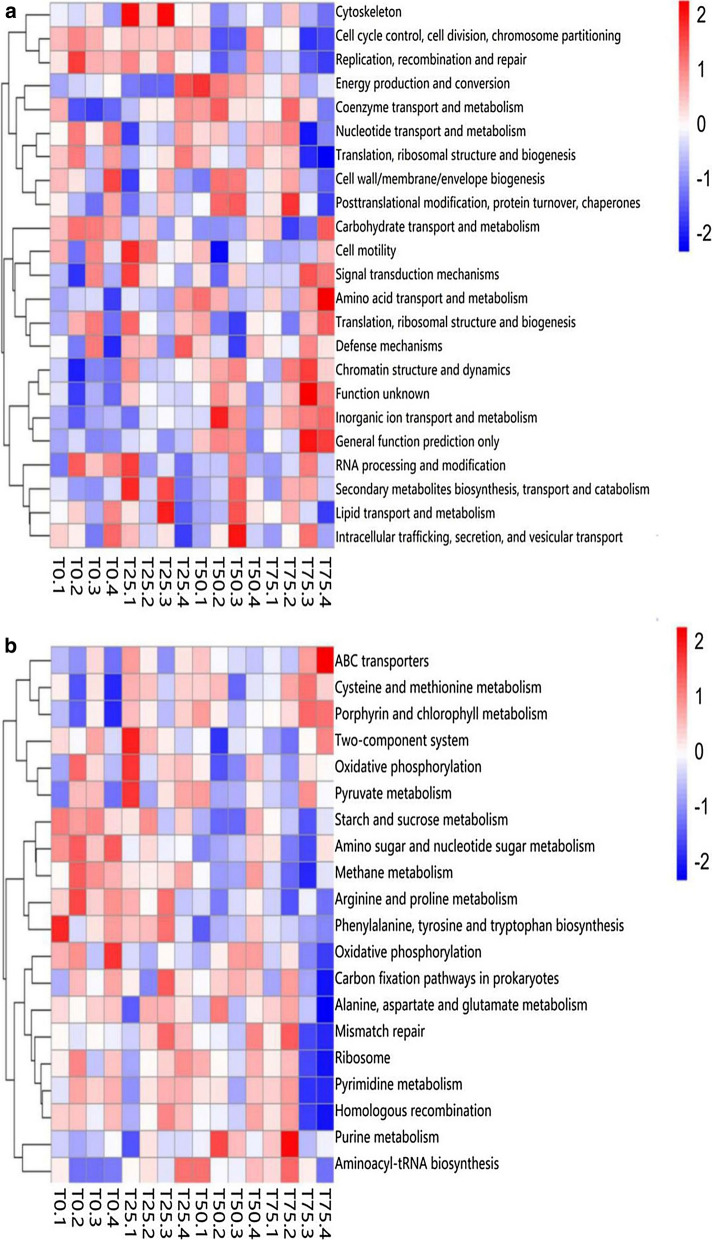
Heatmap of 16S rRNA gene-predicted functional (**a**) and pathway-predicted (**b**) profiles obtained via Phylogenetic Investigation of Communities by Reconstruction of Unobserved States (PICRUSt). T0: BPS replaces 0% of WCS; T25: BPS replaces 25% of WCS; T50: BPS replaces 50% of WCS; T75: BPS replaces 75% of WCS. Numbers 1, 2, 3 and 4 in names of samples refer to individual heifers per set of treatment. BPS, *Broussonetia papyrifera* silage; WCS, whole corn silage

16S function prediction was performed via PICRUSt software, which stores a series of databases. By comparing information with different databases, COG information, KEGG Ortholog (KO) information, and pathway information of differential OTUs can be obtained and matched. The abundance of functional category can be calculated according to the abundance of differential OTUs. The results of 16S function prediction were shown in Fig. 9. As shown in Fig. 9a, with the increaing of BPS content, the abundance of some COG functions, such as Inorganic ion transport and metabolism, Chromatin structure and dynamics, and Coenzyme transport and metabolism, increased; others, like Cell motility, Cytoskeleton, and Replication, recombination and repair, decreased. In this study, a total of 222 pathways were obtained, and they involved in metabolism, genetic information processing, environmental information processing, and cellular processes; and the first 20 abundant pathways were shown in Fig. 9b.

## Discussion

In the process of cell metabolism, the body will produce a large number of free radicals like reactive oxygen species (ROS), which have a strong oxidation ability. These free radicals have strong toxicity and cause damage to biological macromolecules such as carbohydrates, proteins, lipids, and DNA, and finally leading to oxidative stress and the recession of physiological function, immunity, and production performance, thus causing diseases (Thannickal and Fanburg 2000; Gill and Tuteja 2020). In order to respond to the adverse effects of oxidative stress, the anti-oxidative defence system (AOS) release various enzymes such as CAT, GSH-Px, and SOD (Prokić et al. 2018). Superoxide dismutase can convert superoxide anion radical (·O_2_^−^) into hydrogen peroxide (H_2_O_2_), subsequently, H_2_O_2_ is broken into H_2_O by CAT or GSH-Px (Olsvik et al. 2005). Malonaldehyde is the final product of lipid peroxidation caused by ROS, the content of MDA can reflect the degree of lipid peroxidation and cell damage in the body, and the increase of MDA content marks the aggravation of cell damage (Esterbauer et al. 1991; Rio et al. 2005; Castillo et al. 2006). Total antioxidant capacity is a comprehensive indicator to measure the antioxidant capacity in vivo, reflecting the dynamic balance between pro-oxidants and anti-oxidants, as well as the free radical metabolism state (Ghiselli et al. 2000). In this study, the MDA content of T25, T50, and T75 are lower than T0, indicating that BPS can enhance the resistance of lipid peroxidation in experimental heifers.

Interleukins (IL) are a group of cytokines which are mainly secreted by leukocytes, playing important roles in immune response and tissue repair. IL-1β is a pro-inflammatory cytokine which features in inflammatory and infectious diseases, leading immune cell recruitment and bacterial clearance eventually (Zhang et al. 2019). IL-4 is an anti-inflammatory cytokine, which is related to regulating immune cells, cancer and trophic responses (Granja et al. 2009; Shamoun et al. 2018; Oviedo-Boyso et al. 2007) found that when cows develop bacteria-induced inflammation, the levels of pro-inflammatory cytokines like IL-1β, IL-2 and IL-6 in the body increased, and the overexpression of pro-inflammatory factor caused tissue damage. A large number of studies showed that IL-4 and IL-10 can inhibit the secretion of pro-inflammatory cytokines like IL-1β and IL-6 (Cawston et al. 1996; Mak and Saunders 2006; Borghaei et al. 2010). The above findings suggest that an increase in IL-1β or a decrease in IL-4 may indicate inflammation. In the present study, the IL-1β content of T25, T50, and T75 decreased significantly compared with T0; the IL-4 and IgA content of T25, T50, and T75 increased, demonstrating that BPS has the potential to improve immunity of heifers. The immunomodulatory function of *Broussonetia papyrifera* is derived from its bioactive substances, similar reports had been reported in other woody forages like *Moringa oleifera* and *Neolamarckia cadamba* (Pandey and Negi 2016; Valdivié-Navarro et al. 2020).

Corn is a starch-rich feed material. Starch that has not been digested by rumen microorganisms and small intestinal enzymes will enter the hindgut and continue to ferment, leading to the decreasing of fecal pH value (Petri et al. 2019). Superfluous fermentable carbohydrates can cause hindgut acidosis (Sulzberger et al. 2016). In the present study, the fecal pH value of T0 is lower than the other three treatments, presumably because the fermentable carbohydrates content of T0 is the highest among the four treatments. VFAs mainly come from the decomposition of carbohydrates. Rumen microorganisms can convert carbohydrates such as starch, cellulose, and soluble sugar into pyruvate, which can be converted into different VFAs due to different metabolic pathways. Moreover, VFAs play important roles in maintaining the integrity of intestinal epithelial morphology and function (Sato et al. 2009; Missotten et al. 2010). Fecal NH_3_-N is derived from the hydrolysis of amino acids and proteins by proteolytic enzyme and deaminase; and is correlated with N intake strongly (Weiss et al. 2009). In addition, according to another research, when animals ingest diets containing high NDF, a large number of microorganisms related to fiber degradation in the rumen take advantage of NH_3_-N as the main nitrogen source for metabolism, resulting in the decrease of NH_3_-N in the rumen and intestinal track (Hristov and Ropp 2003). In this study, compared with T0, NH_3_-N content in the other three treatments occurred no significant changes. In conclusion, the substitution of WCS for BPS had no significant effect on the intestinal fermentation parameters of heifers.

Gastrointestinal tract is an important habitat for bacteria, intestinal bacteria play important roles in the health and growth of host. Learning the microbial community structure of feces is important for reducing foodborne pathogens through diets changes. In the present study, no significant differences were presented in Shannon index, Ace index, and Chao index, indicating the BPS had no obvious effect on α-diversity, and the result is similar to another two studies. Sun et al. (2017) and Li et al. (2017) fed cows and steers with ensiled *M. oleifera* and ensiled mulberry leaves respectively, finding that there were no conspicuous differences on α-diversity. According to Fig. 1 and 87.46% of OTU in the four treatments is uniform. The results above demonstrate that the component of coarse fodder cannot affect the fecal bacterial community significantly.

On the phylum level, the dominant phyla is *Firmicutes* and *Bacteroidetes*, according to a report, the two phyla are predominant in the large intestine of numerous mammals such as human being, ruminant, pig, and mouse (Ban-Tokuda et al. 2017). *Firmicutes* and *Bacteroidetes* contain various carbohydrate utilizing enzymes, play a vital part in fiber degradation (Xu et al. 2019). The addition of BPS has no effect on the species of dominant phyla in feces, but the relative abundance of dominant phyla can be improved. *Verrucomicrobia* is the third dominant phylum, with the increasing of BPS content, the relative abundance of this phylum improves accordingly. A previous study found that *Verrucomicrobia* has the potential to induce and regulate immunity, it may be a target of intestinal microbial intervention to improve the regulation of immunity (Lindenberg et al. 2019). The increasing of *Verrucomicrobia* may be associated with improved intestinal immunity. On the genus level, the dominant phyla is *Ruminococcaceae_UCG-005*, *Ruminococcaceae_UCG-010*, and *Rikenellaceae_RC9_gut_group*. The result is different from another two studies (Li et al. 2012; Zhao et al. 2017). In addition, the dominants of different treatments in this study present significant difference, indicating diets has a great influence on the genus level of fecal bacterial community of heifers (Kim et al. 2014). According to Li et al. (2018), with the severity of diarrhea, the relative abundance of *Ruminococcaceae_UCG-005*, and *Rikenellaceae_RC9_gut_group* in the musk deer feces decrease, in this study, the relative abundance of above two genera in T25 is higher than that in T0, T50, and T75, indicating that moderate BPS may be able to relieve diarrhea, however, this deserves further study.

LEfSe analysis is an analytical tool for the discovery and interpretation of high-dimensional data biomarker. It emphasizes statistical significance and biological correlation, and was able to look for biomarkers that differed statistically from group to group. In this study, 12 differential OTUs including one class, one order, three families, and seven genera are obtained. *Paeniclostridium* is considered a potential pathogen, it may be related to soft tissue infection and toxic shock (Kim et al. 2017). *Gelria* (*Thermoanaerobacterales* Order, *Thermoanaerobacteraceae* Family) is known to be related to the metabolism of VFAs (FitzGerald et al. 2019). *Roseburia* is a common butyrate-producing bacteria in the intestinal tract (Hatziioanou et al. 2013; Sheridan et al. 2019). *Ruminococcaceae* are common in both rumen and hindgut of ruminants, playing important parts in degrading starch and cellulose (Zhang et al. 2020). In another study, Han et al. (2018) found that *Ruminococcaceae* is related to the balance of Treg/Th17, suggesting that *Ruminococcaceae* may be related to the immune system. Reports and researches about function of the other differential OTUs is limited, however, these differential OTUs can be investigated as biomarkers in depth. As we can see in Fig. 5a, *Gelria* is positively correlated with MDA content, and shows a weak negative correlation with SOD, GSH-Px, and T-AOC, thus, it can be researches as a potential antioxidant biological target. Another thing worth noting is shown in Fig. 5b, *Coprococcus_3* is positively correlated with IL-1β while negatively correlated with IL-4. We have described in the preceding text that IL-1β is a proinflammatory cytokine, and IL-4 can inhibit its secretion. Therefore, it can be speculated that the increasing of *Coprococcus_3* indicates the occurrence of inflammation.

PICRUSt is an analysis method in order to predict the gene function profile of archaea and bacteria based on the measured bacterial genome of 16S rRNA sequence. As shown in Fig. 6a, BPS inhibits carbohydrate transport and metabolism, and promotes amino acid transport and metabolism; the metabolites of carbohydrate and amino acid in the hindgut is VFA and NH_3_-N, respectively. Presumably, the change of bacterial community causes the change of gene function abundance, thereby causing the change of metabolites. As shown in Fig. 6b, the abundance of Methane metabolism decreases with the increasing of BPS content, may indicating BPS can reduce methane production; and this confirm the previous point of view.

In conclusion, this research reveals that replace WCS with a certain proportion of BPS might benefit the gut health of Holstein heifers; and the BPS content has significant impacts on fecal bacterial community. In-depth research, 12 differential OTUs are obtained in the four treatments, and they have correlation with some indicators of serum and hindgut to some extent. These differential OTUs can be researched as potential biomarkers in order to observe the changes in healthy status. Finally, we find that BPS changes the abundance of genes and pathways related to various life activities.

## Data Availability

The sequences in this study were submitted to the Sequence Read Archive (SRA) and a BioProject number PRJNA594421 was obtained.
